# The Sociodemographic Digital Divide in Mobile Health App Use Among Clients at Outpatient Departments in Inner Mongolia, China: Cross-sectional Survey Study

**DOI:** 10.2196/36962

**Published:** 2022-05-19

**Authors:** Li Cao, Virasakdi Chongsuvivatwong, Edward B McNeil

**Affiliations:** 1 Information Technology Department Inner Mongolia Medical University Hohhot China; 2 Department of Epidemiology Faculty of Medicine Prince of Songkla University Songkhla Thailand

**Keywords:** digital divide, mHealth, app, structural equation modeling, client

## Abstract

**Background:**

Mobile health (mHealth) apps have become part of the infrastructure for access to health care in hospitals, especially during the COVID-19 pandemic. However, little is known about the effects of sociodemographic characteristics on the digital divide regarding the use of hospital-based mHealth apps and their benefits to patients and caregivers.

**Objective:**

The aim of this study was to document the cascade of potential influences from digital access to digital use and then to mHealth use, as well as the potential influence of sociodemographic variables on elements of the cascade.

**Methods:**

A cross-sectional survey was conducted from January to February 2021 among adult clients at outpatient departments in 12 tertiary hospitals of Inner Mongolia, China. Structural equation modeling was conducted after the construct comprising digital access, digital use, and mHealth use was validated.

**Results:**

Of 2115 participants, the β coefficients (95% CI) of potential influence of digital access on digital use, and potential influence of digital use on mHealth use, were 0.28 (95% CI 0.22-0.34) and 0.51 (95% CI 0.38-0.64), respectively. Older adults were disadvantaged with regard to mHealth access and use (β=–0.38 and β=–0.41), as were less educated subgroups (β=–0.24 and β=–0.27), and these two factors had nonsignificant direct effects on mHealth use.

**Conclusions:**

To overcome the mHealth use divide, it is important to improve digital access and digital use among older adults and less educated groups.

## Introduction

The term mobile health (mHealth) was coined in 2003 [1] and is defined as health care practice through mobile devices and their apps [2,3]. mHealth apps have been developed for hospitals to allocate and manage their medical care services and to improve patient satisfaction [4,5]. During the COVID-19 pandemic, mHealth apps were used to implement prescreening, tracking cases, and social distancing measures [6-8]. COVID-19 as an extra factor is also exacerbating existing inequalities [9]. Older adults and less educated people have been affected the most by lockdown measures [9,10]. Some hospitals have implemented mHealth-based online appointments and video consultations with health care providers [11,12], instead of traditional register windows and consultation rooms, in order to reduce contacts [13,14]. Vulnerable people benefit the least from these digital solutions [9,10].

**Figure 1:**
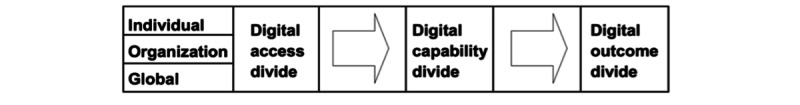
The three-level digital divide framework.

The digital divide is defined as a gap between people who have access to internet services and those who do not [15,16]. The digital divide is a central issue in the world today [17,18] and is described using a three-level framework [19] (Figure 1). Around half the number of people worldwide have access to the internet. Sociodemographic characteristics, particularly age, gender, education, and income, predict internet access and use [7,20].

As of December 2020, Chinese internet penetration had reached 70.4% [21]. Patient satisfaction has become an important indicator for measuring health care quality [22,23] and policy evaluations of health care systems, which directly connect with health services use [24]. mHealth app features affect user satisfaction in various health care scenarios [25,26]. mHealth is a specific area that can be used to examine the digital divide. The Chinese government has tried to implement reforms to reduce waiting times and improve health care and patient satisfaction, including the use of mHealth [27-29]. In 2015, the Chinese government issued an action plan requiring at least 50% of appointments to be online for visiting doctors in tertiary hospitals by the end of 2017 [30]. However, the adoption rate of mobile services for outpatients was low, accounting for only 31.5% in 2019 [31]. Whether mHealth in China has reached its goal is still a subject for debate.

In China, 83% of tertiary hospitals provide online appointments, of which 60% have mHealth services [4]. The majority of mHealth apps were nested into WeChat using an official account or a mini-app [12,32]; WeChat is the most popular social media platform in China, whereas other platforms have been built by local governments, companies, or hospitals. Common mHealth services based out of hospitals are extremely similar to each other. Generally, potential users must first install the mHealth app or subscribe to the WeChat official account, sign up to be a user, then sign in to use the mHealth service. Users can make an appointment with the doctors and pay the fee for clinical tests or medicine [33]. mHealth use is recognized as a fundamental social determinant of health [34], which facilitates access to medical care services and health outcomes [35,36]. Meanwhile, hospital-based mHealth apps can be used by caregivers, who may act as proxy users on behalf of patients to reduce the digital divide [37]. 

Inner Mongolia is a province located in the northern part of China and borders Mongolia and Russia to the north. It is an underdeveloped province [38] containing 49 ethnic minority groups. Traditional Mongolian medicine, traditional Chinese medicine, and Western medicine are well supported by the government and accepted by local citizens [39]. Within this context, it is likely that there is digital divide between different groups of health care users. A study on digital divide among hospital clients in this area could lead to improvement of health care in the future.

**Figure 2:**
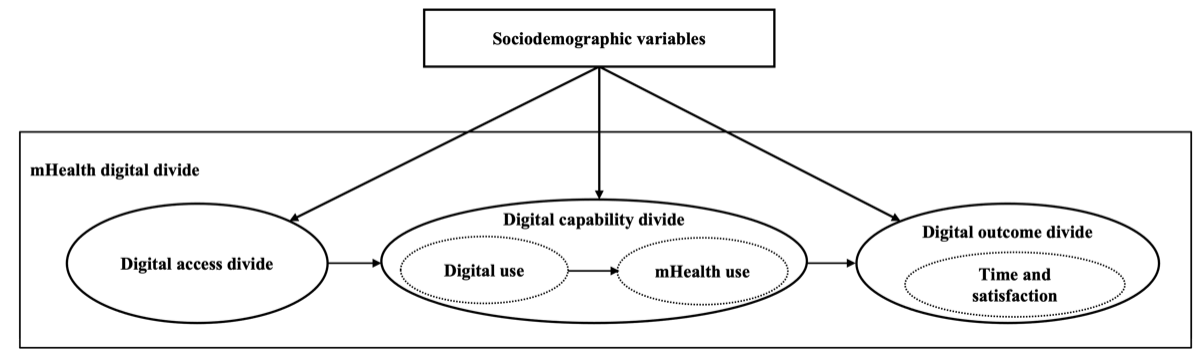
Research and hypothesis model of mobile health (mHealth) digital divide.

To date, few studies have been conducted to analyze the digital divide regarding the use of hospital-based mHealth apps. All previous studies ran regressions to find the potential influence of sociodemographic variables on mHealth use without considering the clients’ digital access and digital use backgrounds. Our framework of evaluation of the digital divide in mHealth covers the whole spectrum, from digital access to digital use, mHealth use, and time and satisfaction when using health care services (Figure 2). Separating the potential influence of sociodemographics on each part of the cascade, with simultaneous evaluation of the flow of potential influence along the cascade, will lead to better understanding and a more appropriate formulation of policy to minimize these digital divides.

## Methods

### Study Design and Setting

A cross-sectional survey was conducted in January and February 2021 in 12 tertiary hospitals across three cities of the Inner Mongolia Autonomous Region. All studied hospitals provide mHealth apps with eHealth codes, appointments with doctors, electronic payment (e-payment), and health record checking.

### Participants

Clients (ie, patients and caregivers) who were visiting the outpatient department for nonemergency care, were aged 18 years or older, and able to speak Chinese were eligible for the study.

### Questionnaire

The questionnaire was created by the research team and reviewed by an epidemiologist from the public health school with mHealth research expertise. The IT department of the Affiliated People’s Hospital of Inner Mongolia Medical University was consulted twice on the amended questionnaire.

### Data Collection Procedure

A study team from the Inner Mongolia Medical University comprised of resident physicians was assembled and trained on the data collection process. The interviewers consecutively contacted clients at a drugstore or outpatient departure areas, explained the study, and asked for their consent to participate in the study. Consenting participants were asked to complete the questionnaire via face-to-face interviews.

### Ethics Approval

This research study was approved by the Office of Human Research Ethics Committee, Faculty of Medicine, Prince of Songkla University, Thailand (REC.63-306-18-1).

### Variables

#### Independent Variables

Independent variables included demographic information (ie, age, gender, and area of residence) and socioeconomic status (ie, educational level, employment status, and household monthly income).

#### Constructing the Mediators

Initially, we created a set of digital activities, including having household internet bandwidth, using a computer, using email, having a smartphone, having the ability to install apps, having wearable devices, using the internet, shopping online, using e-payment, using social media, searching online, using the internet daily, and using the internet for more than 5 years. For mHealth app use, since all apps were similar, we did not specify an mHealth app name in the questionnaire. mHealth app use was a latent intermediary variable, with its value loaded onto a series of observed variables: having mHealth apps, having an eHealth code, making appointments with doctors, attending consultations with doctors, making e-payments for medical care, and checking health records. All of these observed variables were on a “yes or no” binary scale. We then randomly split the data set in half to run exploratory factor analysis (EFA) in order to obtain the constructs; we then analyzed reliability within each factor. Subsequently, the constructs were tested on the remaining half of the data set for validation via confirmatory factor analysis (CFA).

#### Dependent Variables

Dependent variables included time and satisfaction with health care. We selected four indicators related to mHealth use: waiting time, check-in process, medicine withdrawal and payment process, and general satisfaction. These indicators were assessed using the Chinese Outpatient Experience Questionnaire [40], which uses a 5-point Likert scale, ranging from 1 (the worst satisfaction) to 5 (the best satisfaction).

### Statistical Analysis

Data were double-entered into EpiData (version 3.1; The EpiData Association), and analysis was performed using R (version 4.1.0; The R Foundation). Descriptive statistics were used to summarize the characteristics of the clients, namely frequency with percentage for categorical variables and mean with SD for continuous variables.

CFA was used to analyze the correlation matrix among the domains. A multiple-indicator, multiple-cause model (MIMIC) with structural equation modeling (SEM) [41] was used to examine the association between sociodemographic variables, mHealth use, and time and satisfaction with health care. The “polycor” R package was used for polychoric and polyserial correlations of categorical variables [42], the “psych” R package was use for EFA [43], and the “lavaan” R package was used for CFA and SEM [44]. The sample size per hospital was calculated based on the assumption that 38.4% of Chinese adults have an mHealth app [45], using an infinite population proportion formula as follows:




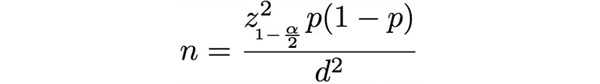




with a 10% error rate (*d*) and a 95% CI (α=.05). A 10% nonresponse rate was also assumed. With these parameters, 102 participants were required to be recruited from each hospital. Due to the effect of COVID-19 on mHealth use, we decided to recruit 200 participants from each hospital. Finally, 2366 participants were included.

## Results

### Sociodemographic Factors

A total of 2115 clients provided valid responses. Their mean age was 43.34 (SD 15.39) years. Other demographic characteristics are summarized in Table 1. The participants were distributed nearly equally between the two genders. Three-quarters resided in an urban area. Almost half of the participants were educated at the tertiary education level. More than half were employed by the government or a company. Their household incomes were also somewhat evenly distributed, with a median of ¥4000 to ¥6000 (a currency exchange rate of ¥1=US $0.15 is applicable), which was deemed to be middle class in China [46].

**Table 1:** Basic characteristics of the participants.

Variable				Participants (N=2115), n (%)^a^
**Sociodemographic variables**				
	Age (years), mean (SD)		43.34 (15.39)	
	**Gender**			
		Male	1007 (47.61)	
		Female	1108 (52.39)	
	**Urban residence**			
		Yes	1630 (77.07)	
		No	485 (22.93)	
	**Educational level**			
		Primary or less	297 (14.04)	
		Secondary	805 (38.06)	
		Tertiary	1013 (47.90)	
	**Employment status**			
		Employed	1166 (55.13)	
		Unemployed	949 (44.87)	
	**Household monthly income (¥^b^)**			
		0-2000	220 (10.40)	
		2001-4000	424 (20.05)	
		4001-6000	456 (21.56)	
		6001-8000	345 (16.31)	
		8001-9999	314 (14.85)	
		≥10,000	356 (16.83)	
**Digital activities**				
	**Have household bandwidth**			
		No	365 (17.26)	
		Yes	1750 (82.74)	
	**Use a computer**			
		No	669 (31.63)	
		Yes	1446 (68.37)	
	**Use email**			
		No	925 (43.74)	
		Yes	1190 (56.26)	
	**Have a smartphone**			
		No	129 (6.10)	
		Yes	1986 (93.90)	
	**Have the ability to install apps**			
		No	584 (27.61)	
		Yes	1531 (72.39)	
	**Have wearable devices**			
		No	1564 (73.95)	
		Yes	551 (26.05)	
	**Use the internet**			
		No	244 (11.54)	
		Yes	1871 (88.46)	
	**Shop online**			
		No	555 (26.24)	
		Yes	1560 (73.76)	
	**Use e-payment^c^**			
		No	429 (20.28)	
		Yes	1686 (79.72)	
	**Use social media**			
		No	548 (25.91)	
		Yes	1567 (74.09)	
	**Search online**			
		No	543 (25.67)	
		Yes	1572 (74.33)	
	**Daily internet use**			
		No	473 (22.36)	
		Yes	1642 (77.64)	
	**More than 5 years of internet use**			
		No	687 (32.48)	
		Yes	1428 (67.52)	
**mHealth^d^ use**				
	**Have mHealth apps**			
		No	792 (37.45)	
		Yes	1323 (62.55)	
	**Have eHealth code**			
		No	682 (32.25)	
		Yes	1433 (67.75)	
	**Make appointments with doctors**			
		No	978 (46.24)	
		Yes	1137 (53.76)	
	**Attend consultations with doctors**			
		No	1876 (88.70)	
		Yes	239 (11.30)	
	**Use e-payment for medical care**			
		No	1102 (52.10)	
		Yes	1013 (47.90)	
	**Health record checking**			
		No	1409 (66.62)	
		Yes	706 (33.38)	

^a^All values are reported as n (%), except for the age variable.

^b^A currency exchange rate of ¥1=US $0.15 is applicable.

^c^e-payment: electronic payment.

^d^mHealth: mobile health.

### Digital Activities

Overall, 88.46% of the participants used the internet, 82.74% had access to the internet at home, and 93.90% had a smartphone. In total, 68.37% of participants used a computer, and 56.26% could use email. Three-quarters of the participants could self-install an app, and one-quarter wore smart wearable devices. Around three-quarters of the participants purchased commodities online, used e-payment, used social media, and performed information-searching online. Most used the internet daily and had been using it for more than 5 years.

### mHealth App Use

Overall, 62.55% of the participants had an mHealth app, 67.75% had an eHealth code, 53.76% could make an appointment to see a doctor, 47.90% used e-payment for health care, 33.38% reviewed their health record on an mHealth app, and 11.30% consulted with a doctor online (Table 1).

### EFA Model of mHealth Digital Divide

From the EFA, validation of the classification of digital activities was performed with a Kaiser-Meyer-Olkin test [47], with a sample adequacy of 0.929 [48], and a Bartlett test of sphericity, which was statistically significant (*χ*^2^_253_=1260.1, *P*<.001) [49]. Based on the parallel analysis, four factors were determined [50]. Due to the nonnormally distributed data, principal axis factoring was used as an appropriate extraction method [51], and oblimin rotation was used as an appropriate oblique rotation method [52]. Five items (ie, “have household bandwidth,” “have wearable devices,” “use social media,” “use e-payment,” and “attend consultations with doctors”) had factor loadings of less than 0.4 [53] or high cross-loadings [54] and were, thus, dropped from the analysis.

Finally, we came up with four domains (ie, factors): digital access, digital use, mHealth use, and time and satisfaction with health care. Details of the EFA are shown in Table 2.

The factor loadings were high, ranging from 0.469 to 0.886. Cronbach α values were all above .8. These four factors explained 62% of the variance. All of these values suggested that our construct was adequate.

**Table 2:** Measurement items and their reliability by exploratory factor analysis.

Factor and items		Loading	Communality	Cronbach α^a^	Proportion of total variance^a^
**Digital access**					
	Have a smartphone	0.717	0.557	.80	0.121
	Use the internet	0.886	0.825		
	Daily internet use	0.542	0.478		
**Digital use**					
	Have the ability to install apps	0.740	0.716	.92	0.212
	More than 5 years of internet use	0.613	0.517		
	Shop online	0.604	0.676		
	Search online	0.696	0.689		
	Use a computer	0.878	0.721		
	Use email	0.881	0.703		
**mHealth^b^ use**					
	Have mHealth apps	0.610	0.533	.86	0.154
	Have eHealth code	0.469	0.373		
	Make appointments with doctors	0.863	0.646		
	Use e-payment^c^ for medical care	0.846	0.771		
	Health record checking	0.710	0.563		
**Time and satisfaction with health care**					
	Waiting time	0.779	0.586	.84	0.133
	Check-in process	0.836	0.728		
	Medicine withdrawal and payment process	0.803	0.658		
	General satisfaction	0.649	0.423		

^a^Values for groups are reported in the row of the top variable of the group.

^b^mHealth: mobile health.

^c^e-payment: electronic payment

### CFA and SEM Model of mHealth Digital Divide

The second part of the data set was used to assess reliability and validity by CFA. The measurement model of digital divide in mHealth app use was adequately measured by associated indicators based on high factor loadings. The correlations among latent variables by CFA are shown in Table 3. The model fitted the data well, according to the following indices: *χ*^2^_129_=528.3, *χ*^2^/*df*=4.095*,* comparative fit index (CFI)=0.940, Tucker-Lewis index (TLI)=0.929, root mean square error of approximation (RMSEA)=0.054 (90% CI 0.049-0.059), and standardized root mean square residual (SRMR)=0.042. The Cronbach α reliability coefficient was greater than .7, and convergent validity based on average variance extracted was greater than 0.5.

A MIMIC model with SEM was investigated for the model of mHealth digital divide by using the weighted least square mean and variance adjusted estimator [55], since most of the variables were categorical. The overall indices of the final SEM model fitted the data well: *χ*^2^_216_=682.6, *χ*^2^/*df*=3.160, RMSEA=0.045 (90% CI 0.041-0.049), SRMR=0.036, CFI=0.949, and TLI=0.938 [53].

**Figure 3:**
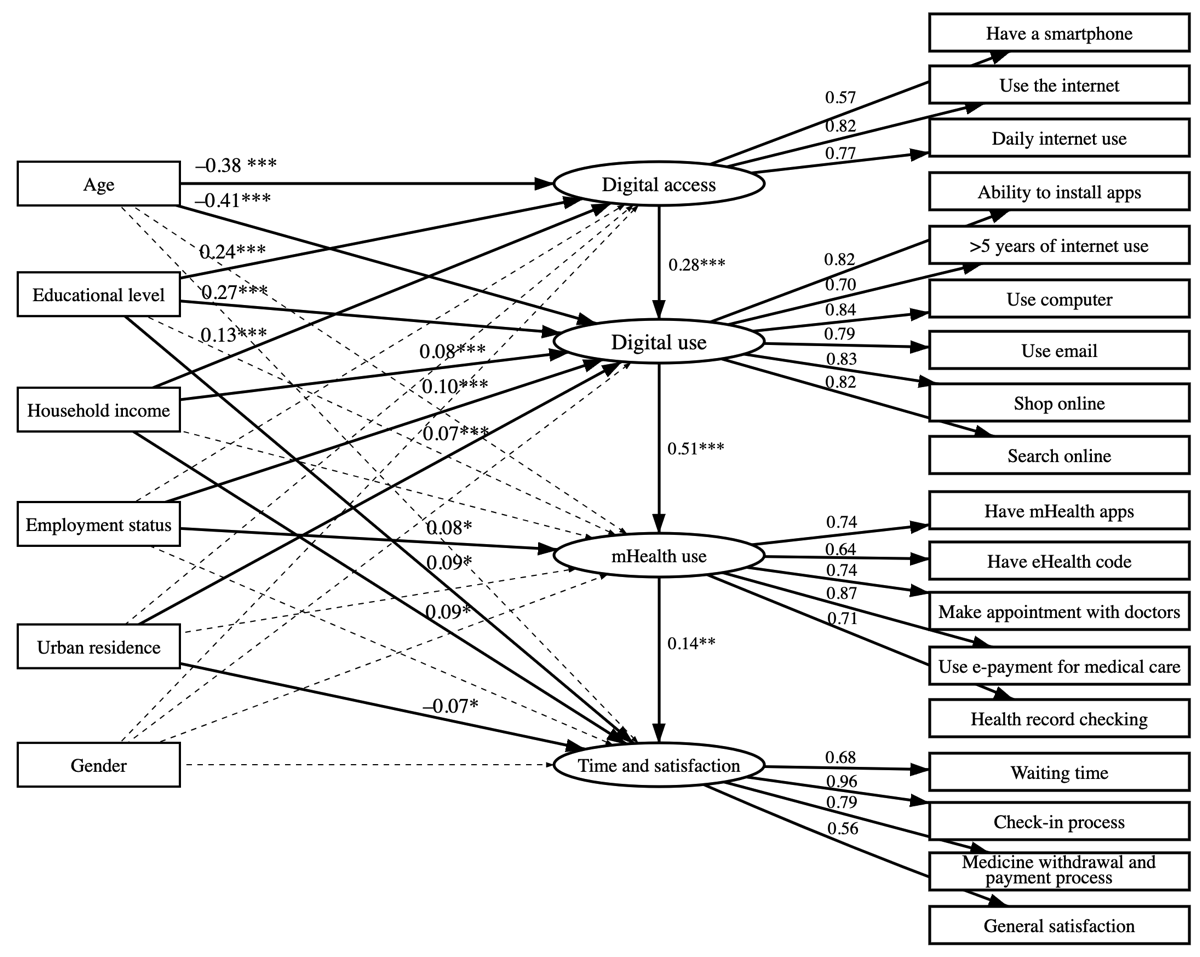
Structural equation modeling for digital divide in mobile health (mHealth). Solid lines represent significant relationships, and dotted lines represent nonsignificant ones; numbers on the lines from sociodemographic variables to latent variables are standardized coefficients, and numbers on the lines from latent variables to items are loadings. ***P<.001, **P<.01, and *P<.05.

Figure 3 shows the regressions of all the paths. The details of the β coefficients and 95% CIs are shown in Table 4. Age was taken as a continuous variable. Although education and household income were initially ordinal categorical variables, we standardized them as continuous variables to suit the SEM. Their coefficients were interpreted as whether these had a dose-response relationship with the outcome. Age and education were strongly associated with digital access and digital use. Income had a low effect on digital access, and income, employment status, and urban residence were weakly correlated with digital use. No significant gender gap regarding these variables was seen. The cascaded coefficients (95% CI) from digital access to digital use, then to mHealth use, and then to time and satisfaction with health care were 0.28 (95% CI 0.22-0.35), 0.51 (95% CI 0.38-0.64), and 0.14 (95% CI 0.05-0.22), respectively. mHealth use, however, had a weakly significant effect on time and satisfaction with health care. mHealth use was not significantly associated with sociodemographic variables, except for employment status, with which it had a weak correlation.

The potential influence of sociodemographic characteristics reflected the level of digital divide. The number of variables and the magnitude of the coefficients were higher for digital use than for digital access and mHealth use. Thus, use divide in our setting was the most important gap.

**Table 3:** Correlation analysis (Pearson r and 2-tailed *P* value) among latent variables by confirmatory factor analysis.

Latent variable				Digital access			Digital use		mHealth^a^ use		Time and satisfaction with health care
**Digital access**											
	*r*	1.000			0.718^b^			0.417^b^		0.159^b^	
	*P* value	—^c^			<.001			<.001		.001	
**Digital use**											
	*r*	0.718^b^			1.000			0.607^b^		0.226^b^	
	*P* value	<.001			—			<.001		<.001	
**mHealth use**											
	*r*	0.417^b^			0.607^b^			1.000		0.231^b^	
	*P* value	<.001			<.001			—		<.001	
**Time and satisfaction with health care**											
	*r*	0.159^b^			0.226^b^			0.231^b^		1.000	
	*P* value	.001			<.001			<.001		—	
Cronbach α			.745			.912			.857		.835
Average variance extracted			0.582			0.639			0.559		0.587

^a^mHealth: mobile health.

^b^The correlation is significant at a significance value of .05 (2-tailed).

^c^Not applicable.

**Table 4:** Regression weights of parameters by the multiple-indicator, multiple-cause model with structural equation modeling.

Link	β coefficient (95% CI)	*P* value
Age → digital access	–0.38 (–0.45 to –0.31)	<.001
Age → digital use	–0.41 (–0.46 to –0.36)	<.001
Age → mHealth^a^ use	–0.04 (–0.15 to 0.06)	.40
Age → time and satisfaction with health care	–0.08 (–0.16 to 0.01)	.07
Educational level → digital access	0.24 (0.15 to 0.32)	<.001
Educational level → digital use	0.27 (0.22 to 0.33)	<.001
Educational level → mHealth use	0.04 (–0.05 to 0.12)	.41
Educational level → time and satisfaction with health care	0.09 (0.00 to 0.17)	.04
Household income → digital access	0.13 (0.06 to 0.19)	<.001
Household income → digital use	0.08 (0.04 to 0.12)	<.001
Household income → mHealth use	0.02 (–0.05 to 0.08)	.62
Household income → time and satisfaction with health care	0.09 (0.02 to 0.17)	.01
Employment status → digital access	0.03 (–0.02 to 0.09)	.20
Employment status → digital use	0.10 (0.07 to 0.14)	<.001
Employment status → mHealth use	0.08 (0.02 to 0.15)	.01
Employment status → time and satisfaction with health care	0.003 (–0.07 to 0.07)	.94
Urban residence → digital access	–0.02 (–0.09 to 0.05)	.53
Urban residence → digital use	0.07 (0.03 to 0.11)	<.001
Urban residence → mHealth use	–0.04 (–0.09 to 0.02)	.20
Urban residence → time and satisfaction with health care	–0.07 (–0.14 to –0.01)	.03
Gender→ digital access	0.06 (0.00 to 0.11)	.06
Gender → digital use	–0.01 (–0.05 to 0.02)	.41
Gender → mHealth use	–0.05 (–0.11 to 0.00)	.07
Gender → time and satisfaction with health care	0.006 (–0.06 to 0.07)	.85
Digital access → digital use	0.28 (0.22 to 0.35)	<.001
Digital use → mHealth use	0.51 (0.38 to 0.64)	<.001
mHealth use → time and satisfaction with health care	0.14 (0.05 to 0.22)	.002

^a^mHealth: mobile health.

## Discussion

### Principal Findings

We confirmed the framework of digital divide in mHealth app use. Our subjects were mostly educated and middle class, with good experience in internet use and other digital media. Between one-half to two-thirds were using basic mHealth features. Sociodemographic factors had stronger potential influences on digital use than on digital access; they also had the least direct effect on mHealth use and time and satisfaction with health care. However, mHealth use was potentially influenced by digital use. Time and satisfaction with health care, on the other hand, was only weakly associated with mHealth use.

The data from this study identified a cascade of potential successive influences, where digital access influenced digital use, which then influenced mHealth use. mHealth use was determined by digital access and use. Similar to our study, a study by Tirado-Morueta et al [20] found that there was an indirect potential influence pathway from physical access to operative use and expressive informative use of the internet; ignoring this intermediary and simple running regression that predicted mHealth use from sociodemographics would lead to a misinterpretation of the results.

Among the sociodemographic variables examined, age and educational level were the stronger potential influencing variables. Both had direct independent influence on digital access and use, but had no direct effect on mHealth use; in addition, educational level had little effect on time and satisfaction with health care. Thus, their effects occurred in the early part of the digital chain. Based on this potential pathway, assistance for older adults and less educated public clients would need to start with improvements in access and use of the internet in general, as well as mobile facilities, such as email, social media, and online business. Experience with these will make mHealth use easier for them.

Sociodemographic variables had a stronger potential influence on digital use than on digital access. This may reflect that the use divide was due to lifestyle differences more than it was due to inequity problems [56]. The cost of digital access in China was relatively small (around US $15/60 Mbps or more per month [57]) and, hence, did not contribute much to digital access inequalities. On the other hand, certain sociodemographic groups, such as youth and upper-class people, choosing or needing to use digital technology is due to their lifestyle [58]. The small but significant correlation (β=0.28) between digital access and digital use may, in fact, reflect a noncausal relationship.

With the use of SEM, our findings on the effects of sociodemographic variables were different from those of other studies using one-step regression [59,60]. These other studies showed that sociodemographic variables potentially influenced mHealth use, but they missed the fact that the effect passed through digital use. Their findings would indicate the emphasis to improve mHealth use among the underprivileged. Our findings, on the other hand, imply that improved general digital use would be a more natural way to empower these groups of clients. This will make it easier and probably more effective to introduce mHealth to them. To reduce the existing digital divide among hospital clients, the hospital administration should provide special services or appropriate education to assist clients in making better use of mHealth apps.

According to another study in China, mHealth was effective in reducing patient waiting times and increasing patient satisfaction in tertiary hospitals [26]. Another study found that waiting times for consultations and prescription filling reduced, resulting in increased outpatient satisfaction with pharmacy services [27]. Moreover, our study validated the marginally significant effect of mHealth use on shorter waiting times and improved satisfaction. This indicates that mHealth app use cannot adequately explain shorter waiting times and increased satisfaction. This indicates a need for further study. Additionally, since mHealth app use in hospitals is in its infancy, mHealth apps must be improved in terms of design and marketing based on existing digital use to increase their use and provide benefits to clients [33].

### Limitations

This was a cross-sectional study. One may argue that the causation proposed in the SEM was limited by temporal sequence and may not be valid. We argue that sociodemographic variables are long-term values and do not vary much over time, whereas digital divide in these domains only comes after. Mobile apps were developed nearly a decade after the wide use of the internet began, and hospital-based mHealth is the most recent development. Therefore, our proposed pathway may not be flawed in terms of temporal sequence. The current stage of mHealth development in our setting is changing continuously. Thus, further studies may produce different results.

### Conclusions

In order to close the mHealth use divide, it is important to improve digital access and digital use among older adults and less educated groups.

## Abbreviations

ASEAN: Association of Southeast Asian Nations
CFA: confirmatory factor analysis
CFI: comparative fit index
EFA: exploratory factor analysis
e-payment: electronic payment
mHealth: mobile health
MIMIC: multiple-indicator, multiple-cause model
RMSEA: root mean square error of approximation
SEM: structural equation modeling
SRMR: standardized root mean square residual
TLI: Tucker-Lewis index
